# Primary health care nursing students’ knowledge of and attitude towards the provision of preconception care in KwaZulu-Natal

**DOI:** 10.4102/phcfm.v11i1.1916

**Published:** 2019-11-12

**Authors:** Winifred C. Ukoha, Makhosi Dube

**Affiliations:** 1 School of Nursing and Public Health, University of KwaZulu-Natal, Durban, South Africa

**Keywords:** primary health care, nurses, preconception care, pre-pregnancy counselling, knowledge, attitude

## Abstract

**Background:**

Sub-Saharan African countries have been the worst affected by the high incidence of maternal and child mortality rates and HIV/AIDS (human immunodeficiency virus/acquired immunodeficiency syndrome) pandemic. Preventive care is the area that requires serious attention as a lot of maternal and child morbidity and mortality can be averted through rendering comprehensive care to women of child-bearing age. Preconception care (PCC) is recognised as an important factor in improving pregnancy outcome; yet, most primary health care (PHC) nurses lack the necessary resources to render PCC.

**Aim:**

To describe the PHC nursing student’s knowledge of and attitude towards the provision of PCC.

**Setting:**

Higher Education Institution that offers PHC programme at six different sites to nurses working in the PHC clinics in the province.

**Methods:**

A quantitative, non-experimental, descriptive study design was used. The total population from three sites selected, based on their geographical location were all invited to participate in the study. Questionnaire was used to collect data which was subsequently analysed using the Statistical Package for Social Sciences (SPSS) version 24.

**Results:**

The response rate was approximately 85% (*n* = 138). The respondents have practised in the PHC clinic for more than 1 year. Study centre, age and area of employment were found to be predictors of knowledge, but no direct association was found between the demographic factor and attitude. Furthermore, a significant difference was found between knowledge and age, and between the area of employment and attitude.

**Conclusion:**

PHC nursing students were knowledgeable and had a favourable attitude towards PCC, but the absence of PCC resources in many practices has hindered them to a greater extent. It is recommended that for proper implementation of PCC to occur, health care workers should be provided with the necessary resources.

## Introduction

Preconception care (PCC) or pre-pregnancy care according to World Health Organization^1^ is ‘the provision of biomedical, behavioural and social health interventions for women and couples before conception occurs’. The aim of this care is to improve the health status of individuals by reducing behaviours and environmental elements that contribute to poor pregnancy outcomes.^2^ The World Health Organization (WHO) reports purported that intervening after conception has occurred is usually too late in reducing risk factors which might affect the mother and her unborn child.^1^

Sub-Saharan Africa has been identified to be the area worst affected by the high rate of maternal and child mortality and morbidity rates.^3^ According to the report published by the WHO in 2014, 289 000 maternal deaths occurred globally in 2013; 99% of them were from low and middle-income countries. Over two-thirds (60%) of all these maternal deaths occurred in sub-Saharan Africa. Still, the likelihood of a 15-year-old girl in sub-Saharan Africa dying due to complications related to childbirth is as high as 1:37, when compared to 1:3400 in developed countries. On the other hand, it was projected that in sub-Saharan Africa, 1:12 children die before reaching the age of five, as compared to 1:147 seen in the developed countries.^3^

The South Africa’s 2010–2013 ‘National Committee on Confidential Enquiries into Maternal Deaths’^4^ reports recognised non-pregnancy related infections, obstetric haemorrhage and complications of hypertension in pregnancy as the three conditions that contribute mainly to the preventable maternal deaths. The above-named conditions contribute to 66.7% and 56.8% of the possible and probable avoidable maternal deaths in South Africa and KwaZulu-Natal (KZN), respectively.^4^

Even though many initiatives have been undertaken both globally and nationally to address this issue, the majority of them are not tackling the problem at the grassroots level, which is preconceptionally. Preventive care in health is the area that requires serious attention as a lot of maternal and child morbidity, and mortality can be averted through rendering comprehensive holistic care to reproductive-aged women.^5^ Recently, there has been a call on governments to decrease the amendable risk factors for non-communicable diseases and the core social determinants of diseases by 2020.^5^

To reach the sustainable development goal (SDG) 3,^6^ especially target 3.1 and 3.2 which aims by 2030 to ‘reduce the global maternal mortality ratio to less than 70 per 100 000 live births and to end preventable deaths of new-borns and children under five’ respectively, much greater effort is needed from all the stakeholders. This effort is especially needed towards the preventive aspect; hence United Nations Children’s Fund (UNICEF) has encouraged an acceleration of the pace of progress that targets child survival, especially in high mortality areas in sub-Saharan Africa.^7^

## Background

Preconception care has been advocated as a major intervention to decrease maternal and child mortality.^8^ It is noteworthy that PCC aims to optimise maternal and child health, which is of great importance to adolescents and men and not just prospective parents. Studies have shown the significance of preconception care and the existing interventions.^9,10,11^ PCC has been proven to help prevent pre-term deliveries for all women; inhibit pregnancy in adolescent years and unintended pregnancy; optimise pre-pregnancy weight; promote healthy nutrition which includes the intake of supplements; screening, identifying and handling mental health; and intimate partner violence. It also promotes the vaccination of children and adolescents, prevents and treats sexually transmitted infections including HIV/AIDS (human immunodeficiency virus/acquired immunodeficiency syndrome) screening; diagnoses and manages chronic disease; promotes ending of tobacco use and restricts contact to second-hand smoke.^9^ The problem of obesity is very pronounced in South Africa. Both obesities of mothers and fathers have been recognised to effect the health of the child and contribute to other risk factors in the mothers.^12^ Pre-pregnancy excess weight increases the risk of hypertensive disorders of pregnancy, preeclampsia and gestational diabetes.^13^ Preconception care services are suggested by different stakeholders and studies as a valuable tool to meet the 90 90 90 global target by ensuring that those who are negative remain negative and those who are positive have their viral load suppressed, before conception occurs, so as to decrease the likelihood of infecting the unborn child.^14,15,16^ Teenage pregnancy and child-bearing are among the biggest contributing factors to high maternal and child morbidity and mortality rates around the world.^17^ Teenage pregnancy is a problem in the South African society, and the rendering of preconception services to all women of reproductive age has been reported as a means of reducing the incidences of both teenage and other unplanned pregnancies.^18^ Globally, complications due to pregnancy are the primary cause of death among women between the ages of 15–19 years. Therefore, interventions to delay pregnancy among this group will subsequently reduce the maternal mortality rate. Studies conducted in the United States reveal that strategies that promote preconception health among this group are needed.^17^ It was found that preconception care should start in adolescent years and continue as inter-conception care to give chances for positive behavioural modifications to happen before pregnancy can occur.^10^

## Problem statement

Preconception care is not well-implemented in South Africa and many other countries worldwide;^19^ despite the agreement that to improve pregnancy outcome, a continuum of care should be delivered from pregnancy, delivery, the postnatal period, infancy and childhood, adolescence and on to adulthood.^20^

The knowledge of health professionals and women about preconception is low globally, and there is a confusion among health workers about who is responsible for the provision of PCC.^21^ The knowledge of the content of PCC guidelines is also a problem among primary health care workers as a Brazilian study reveals an awareness of the presence of a protocol but uncertainty with regard to its content.^22^ Moreover, studies have revealed that the primary health care providers’ knowledge and implementation of PCC protocols toward safer conception among HIV-infected women are inadequate,^23^ resulting in high incidences of both horizontal and vertical transmission of HIV and underutilisation of services by women, as well as poor adherence.^24^ The number of new HIV infections is not reducing as they should be, as many women (about 3%) are still seroconverting during pregnancy in South Africa.^25^ This high incidence of HIV seroconversion during pregnancy in South Africa has been mentioned to be the major cause of the failure of prevention of mother-to-child transmission of HIV.^26^ Notwithstanding the level of health education and counselling being given to women, there is still an increased level of unplanned pregnancy among the general population and also among women living with HIV (33% vs. 50%),^27^ which is indicative of a lack of knowledge about PCC and lack of provision of PCC by health care workers.^23^

Although the primary health care (PHC) nurses are at the forefront of rendering preventive interventions, both in the primary health care and in the community settings, PCC is not always provided to women in the PHC setting and women find that health professionals scarcely discuss the availability and need for PCC with them.^28^ There is, however, the paucity of studies that specifically examined PHC nurse’s knowledge and attitudes regarding PCC,^29,30^ especially in developing countries, which is the focus of this study. Additionally, much literature on the topic of discussion exists only in developed countries, and the few studies that are found in the African context are not very specific, but PCC is rather incorporated into other services.^8,31^ Hence there is scarce literature regarding PCC and counselling in the context of Africa. Moreover, many studies on PCC around the world did not target primary health care nurses in isolation, therefore, they were studied together with other primary health care workers. Therefore, investigation of the knowledge and attitudes of PHC nurses is very necessary.

## Research methodology

### Research design

A non-experimental, descriptive, and cross-sectional survey was employed for this study. Through quantitative descriptive studies, a researcher discovers new meaning, describes what is known, determines the frequency with which something occurs, and categorises the information using a predetermined instrument.^32^ This method gives a broader explanation of the knowledge and attitude of PHC nursing student towards the provision of PCC.

### Setting

The research setting for this study was a higher education institution (HEI) in KZN. This study targeted a programme that uses a decentralised mode of teaching in PHC. This primary health care programme is offered at six different sites around KZN to nurses who are already working in the PHC clinics in the province. For this study, three sites under the patronage of the selected HEI were considered, namely Durban, Pietermaritzburg, and Empangeni. These three settings were selected based on their geographical location and characteristics (urban, semi-urban and rural), and accessibility.

### Study population and sampling strategy

The three selected sites have a population of 163 PHC nursing students. Convenience sampling method was used to enrol PHC nursing student from the selected sites who met the criteria, who had agreed to participate in the study. All the PHC nursing students available from the selected sites were asked to participate in the study but 138 out of 163 participated in the study. The minimum sample size required in a population of 200, to achieve a 95% level of confidence and 5% margin of error according to Leslie Kish was 134.^33^ Therefore, a sample size of 138 for a population of 163 is considered very adequate for this study.

The inclusion criterion considers all nurses enrolled in the PHC programme and registered with South African Nursing Council (SANC) as general, community and midwifery nurses, and were willing to participate in the study. All those who had de-registered or were on long sick leave or those that did not practice in a primary health care setting or did not render services to women in the reproductive age and those other under the age of 18 were excluded from the study.

### Research Instrument and validity

The self-administered questionnaire was used to collect data. The instrument was adapted from studies on PCC conducted in the United Kingdom and the Netherlands.^29,30^ It consisted of three sections A to C. Section A was the demographic data, B was composed of the two knowledge parts and C was the attitude. The two instruments were merged to form the knowledge and the attitude section of the instrument, while the researcher added the demographic section. The content and face validity of the instrument were assessed through extensive literature review and in line with the study objective and with the help of the supervisor. The study instrument was pretested by means of a test-retest method to measure the stability and reliability of the instrument over time. The test-retest involved five PHC facilitators who also work in the PHC setting and they were not included in the main study. The ‘Cronbach’s alphas’ for the various sections of the questionnaire were as follows: knowledge: 0.85 and attitude: 0.70.

### Data collection procedure

The study data were collected in October 2017 at the three centres. Consent form, together with information sheet with details of the study, was given to the participants to obtain an informed consent. Questionnaires were subsequently handed out to those who consented to the study, and they were guided on how to complete it. The completed questionnaires were returned at the participant’s convenience.

### Data analysis

Responses from the questions were coded numerically by assigning numbers to the responses given by the respondents to enable analysis of data. ‘Statistical Package for Social Sciences’ (SPSS) version 24 was used to capture and analyse the collected data. The knowledge of PCC as a whole was measured using 10 items on five-point Likert scale, which ranged from strongly disagree to strongly agree. The attitude of PCC was also assessed using 12 items on a five-point Likert scale. The strongly disagree was scored as one and strongly agree scored as five in a positive statement whereas the score was reversed in negative statements. The knowledge and attitude score were further computed to come up with a composite score where respondents who scored below the 75th percentile were categorised as not knowledgeable or with unfavourable attitude while those who scored above the 75th percentile were categorised as knowledgeable or with a favourable attitude. Descriptive statistics, mainly percentages and frequencies, were used to describe and synthesise data, while frequency tables were used to display the frequency of data. The ‘Kruskal–Wallis test’, ‘Mann-Whitney U test’, ‘Fishers exact test’ and ‘Pearson chi-squared test’ was used to test if the distribution of knowledge and attitude differed across the nurses’ social demographic characteristics. A Chi-square test was used to ascertain the independence of knowledge and attitude of nurses towards PCC. As some of the chi-squared test was significant, ‘Logistic Regression’ was further used to identify factors influencing knowledge level. The level of significance was set at 0.05. Furthermore, the crude odd ratios (COR) and adjusted odds ratios (AOR), respectively, were used to explain the relationship between knowledge and the demographic profile of the study population.

Results from the univariate logistic regression model (crude odds ratio) informed the profile of variables to be included into the multivariate logistic regression model. The analysis was all made fixing the confidence interval (CI) at 95%

### Ethical considerations

Ethical clearance for the study was obtained from the University of KwaZulu-Natal (Ethical Committee protocol reference number HSS/0994/017M). This was done to protect the rights of the respondents. Gate keeper’s permission was sought from the registrar and the dean of the HEI before conducting the study. The respondents gave their consent before the questionnaires were distributed to them, and all ethical considerations were maintained, especially anonymity and confidentiality.

## Results

Out of 163, 138 questionnaires distributed were returned giving a response rate of approximately 85%.

### Sociodemographic data

In this study, of 138 PHC nurses, the majority (83.3%) were female. Approximately 47% were aged between 31 and 40 years, and only 8.7% were aged 51 years and above. They were mainly African (92.8%) and 58.7% were married.

For the years of experience, 80.4% had worked from 1 to 5 years, while 19.6% had worked for more than 6 years. The employment area was divided among 83.3% for the public clinic, 12.3% for the municipal clinic, and 4.3% for non-governmental organisation (NGO) and others. For the study centre of the respondents, 38.4% were from Durban, 29% were from Pietermaritzburg and 32.6% were from Empangeni.

### Knowledge of preconception care

The findings of the respondent’s knowledge of PCC showed that on a more favourable note, the majority of the respondents 88.4% (*n* = 122) agreed that PCC can lead to better pregnancy outcome, while 92% (*n* = 127) agreed, that PCC could reduce the incidences of unplanned and unwanted pregnancy. Similarly, a further 92.8% (*n* = 128) agreed that PCC could reduce maternal and child mortality rate and 89.9% (*n* = 124) agreed PCC is for all women of child-bearing age. Most of the respondents 84.1% (*n* = 116) agreed, that PCC could reduce the incidences of HIV PCR positive and more than two-thirds 71.8% (*n* = 99) agreed that PCC could reduce the chances of acquiring HIV among serodiscordant couples while 42.8% agreed that there is little evidence base for PCC. On a less favourable note, more than two-thirds of the respondents 68.1% (*n* = 94) disagreed that PCC would not change the patient’s risk profile and a further majority 81.2% (*n* = 112) disagreed that PCC should only be offered to women with high risks. On the other hand, 44.9% (*n* = 62) were uncertain regarding the absence of PCC policy in South Africa, while 39.2% (*n* = 54) had disagreed and 15.9% (*n* = 22) agreed.

The knowledge about PCC had 10 items; thus, the possible minimum score was 10, and the maximum score was 50. The minimum score for the respondents was 28, and the maximum score was 50; *M* = 39.7 and s.d. (standard deviation) = 4.8. On grouping of the scores, with a score of less than 39 indicating not knowledgeable and 40–50 knowledgeable, the majority of respondents 55.0% (*n* = 76) fell within the knowledgeable group while 45.0% (*n* = 62) were in the not knowledgeable group. Hence, on average, the result indicated that the participants was knowledgeable about PCC.

### Predictors of good preconception care knowledge and favourable preconception care attitude

The knowledge of PCC was compared with the demographic factors of the respondents to assess the predictor of good knowledge. As shown in Table 1, the knowledge of PCC was compared between the three study centres. Urban students’ knowledge of PCC was compared with their counterparts in semi-urban and rural areas. The AOR for semi-urban was 2.6 (95% CI 0.9–6.7), while for the rural population was 5.3 (95% CI 1.9–14.5). Students from rural areas are 5.3 times more likely to be knowledgeable about PCC as compared to urban students. Also, students from semi-rural were 2.6 times more likely to be knowledgeable about preconception care as compared to urban students.

**TABLE 1:** Logistic regression analysis showing the respondents knowledge towards preconception care.

Factor	PHC Nursing Students’ Knowledge of PCC				*p*	COR	95% CI	*p*	AOR	95% CI
	Knowledgeable		Not knowledgeable							
	*n*	%	*n*	%						
**Study centre**										
Urban	24	45.3	29	54.7	0.057	1	-	1	1	-
Semi-urban	21	52.5	19	47.5	0.49	1.3	0.5–3.0	0.05	2.6	0.9–6.7
Rural	31	68.9	14	31.1	0.02	2.7	1.1–6.1	0.001	5.3	1.9–14.5
**Age**										
20–30	17	53.1	15	46.9	0.049	1	-	1	1	
31–40	35	53.8	30	46.2	0.9	1.0	0.4–2.4	0.7	0.8	0.3–2.0
41–50	21	72.4	8	27.6	0.12	2.3	0.7–6.7	0.5	1.5	0.4–4.8
51+	3	25.0	9	75.0	0.15	0.3	0.0–1.2	0.05	0.2	0.04–1.0
**Employment area**										
Public clinic	61	53.0	54	46.9	0.11	1	-	1	1	-
Municipal clinic	13	76.5	4	23.5	0.08	2.9	0.8–9.3	0.005	7.3	1.8–29.3
NGO/others	2	33.3	4	66.7	0.4	0.4	0.0–2.5	0.5	0.5	0.07–3.6
**Gender**										
Male	12	52.2	11	47.8	0.821	-	-	-	-	-
Female	64	55.7	51	44.3	-	-	-	-	-	-
**Race**										
Black	69	53.9	59	46.1	0.386	-	-	-	-	-
Indian	4	57.1	3	42.9	-	-	-	-	-	-
Mixed race	3	100.0	0	0.0	-	-	-	-	-	-
**Religion**										
Christian	68	54.8	56	45.2	1.000	-	-	-	-	-
Islam	2	66.7	1	33.3	-	-	-	-	-	-
Hindu	2	50.0	2	50.0	-	-	-	-	-	-
Traditional	4	57.1	3	42.9	-	-	-	-	-	-
**Marital status**										
Single	45	55.6	36	44.4	0.971	-	-	-	-	-
Married	28	53.8	24	46.2	-	-	-	-	-	-
Divorced	2	50.0	2	50.0	-	-	-	-	-	-
Separated	1	100.0	0	0.0	-	-	-	-	-	-
**Years of experience**										
1–5	61	55.0	50	45.0	0.319	-	-	-	-	-
6–10	11	55.0	9	45.0	-	-	-	-	-	-
11–20	4	80.0	1	20.0	-	-	-	-	-	-
21 and above	0	0.0	2	100.0	-	-	-	-	-	-

PHC, primary health care; PCC, preconception care; COR, crude odd ratios; AOR, adjusted odds ratios; CI, confidence interval; NGO, non-governmental organisation.

The knowledge level as compared across the age groups with ages 20–30 as the reference group revealed that ages 31–40 are 20% less likely to be knowledgeable about PCC and ages 41–50 were 1.5 times more likely to be knowledgeable while those above 50 years of age were 80% less likely to be knowledgeable about preconception care. The knowledge of the respondents was compared across their employment areas with those in the public clinic as the reference group showed that those in the municipal clinics are 7.3 times more likely to be knowledgeable and those working for the NGO’s and others not mentioned are 50% less likely to be knowledgeable. There was no significant association between gender, race, religion, marital status, years of experience and knowledge levels.

### Attitude towards preconception care

The results revealed that most respondents are on a less favourable note; 45.7% (*n* = 63) disagreed that a dedicated clinic for PCC is a luxury service, while 49.2% (*n* = 68) disagreed that a hospital setting was the best place to provide PCC, and interestingly, 58.7% (*n* = 81) disagreed that there was not enough time to provide. In the same vein, 47.8% (*n* = 66) disagreed that as PHC nurses they did not have enough skills to offer PCC, and more than two-thirds of the respondents 82.6% (*n* = 114) disagreed that PHC nurses were not the best people to offer PCC and more than two-thirds 69.6% (*n* = 96) disagreed that initiating the talk about pregnancy wishes was uncomfortable. Similarly, 57.3% (*n* = 79) disagreed that PCC, without women asking for it, was objectionable. On the favourable note, the majority of the respondents 93.5% (*n* = 129) agreed that PCC is an important health issue for women of child-bearing age and 47.1% (*n* = 65) agreed that in their practice, population planning for pregnancy does not often happen. The results further showed that more than half of the respondents 52.2% (*n* = 72) agreed that PCC was a high priority in their workload while 84.8% (*n* = 117) agreed that they preferred to deal with risk factors before pregnancy rather than in pregnancy and 82.6% (*n* = 114) agreed that with PCC they could do something extra for their patients.

The attitude level had 12 items, thus the possible minimum score was 12 and the maximum score was 60. The minimum score for the respondents was 28 and the maximum score was 55 with *M* = 42.9 and s.d. = 5.17. On grouping of the scores, with a score of less than 35 indicating unfavourable attitude and 36–60 indicating favourable attitude, the majority of respondents 92.8% (*n* = 128) has a favourable attitude, and 7.2% (*n* = 10) has a less favourable attitude towards PCC. Hence, on an average, the result indicates that the participant has a favourable attitude towards PCC.

### Difference and association of demographic with knowledge and attitude

Non-parametric testing Kruskal Wallis and Mann-Whitney U were used for difference and associations between the demographic variables with knowledge and attitude. The Kruskal Wallis test revealed a statistically significant difference between the age of the nurses and their knowledge of PCC, *p* = 0.004. There was also a statistical difference found between employment area and attitude, *p* = 0.005. However, no difference was found between knowledge of PCC, attitude and other demographic variables. Mann-Whitney U tests revealed a statistically significant association between knowledge and attitude, with a *p*-value of 0.000.

## Discussion

The ultimate aim of PCC is short- and long-term improvements in maternal and child health outcomes.^1,18^ The results of this study found that a great percentage of the respondents (88.4%) agreed that PCC could lead to better pregnancy outcomes. This is also consistent with a PCC study among midwives^30^ where the majority of them agreed that it can lead to better pregnancy outcomes. Preconception care should be provided to all reproductive-aged women irrespective of their risk profile, but those with a high-risk profile should not be neglected.^34^ Therefore, the majority (81.2%) of the study respondents had disagreed that PCC should only be offered to women with high risks. Likewise, the study among midwives^30^ reported that more than 80% of them disagreed that PCC should only be offered to women with high risks. The results of this study showed that 92% of the respondents agreed that PCC could reduce the incidences of unplanned and unwanted pregnancy. This echoed the findings of a systematic review and a study among women living with HIV^35,36^ were PCC were showed to reduce the incidences of unplanned pregnancy.

There are conflicting reports about nurses and primary health care workers’ knowledge of preconception care. Several studies have revealed a low level of knowledge regarding PCC services,^21,23,37,38^ while a study in the United States^39^ reported that health care workers had a high knowledge regarding specific PCC interventions. However, the results of the present study portrayed that most respondents (55%) were knowledgeable about PCC. These results are almost similar to the previous study on PCC by Sattarzadeh, Farshbaf-Khalili^40^ with a mean score of 73.21 and ±11.83. Factors such as availability of PCC resources, working in public hospital, use of smartphone and practice of PCC have been cited to predict the knowledge of PCC.^41^ Whereas in this study, study centre, age and employment area are predictors of knowledge.

Kachoria and Shawe^34,42^ observed that in many countries, preconception guidance was usually contained within the pregnancy guidelines. Not surprisingly, 44.9% had been uncertain, and 39.2% had disagreed that there is no PCC policy in South Africa. This is because currently South Africa does not have a PCC policy but has some guidance in its maternity guidelines.^43^ Testing and the management of HIV, especially for the prevention of vertical and horizontal transmission, are components of PCC services.^1,44^ Similarly, most respondents (84.1%) also agreed that PCC could reduce the incidence of HIV PCR positive, and a further two-thirds (71.8%) agreed that PCC could reduce the chances of acquiring HIV among serodiscordant couples. This is consistent with the observations in the US and South Africa among people living with HIV.^44,45^ It is also consistent with the recommendations about the goal of PCC in serodiscordant relationships.^8^ Interestingly, the findings of this research show that more than a quarter of the respondents 42.8% had agreed, that there was little evidence base for PCC. This is contrary to the reports of Goodfellow,^46^ who observed that there is a wide range of available PCC interventions with evidence of effectiveness, although some are with a more stronger evidence base than others. Although knowledge among healthcare worker on implementations and protocols about PCC interventions has been reported to be low,^22,23^ their general knowledge about advice and screenings required during preconception services were not.^39^

The results of their attitude dimensions revealed that most respondents had favourable attitude towards preconception care. These results were in line with previous literature on PCC^30,47^ where health workers also possess favourable attitude. Other studies also reported positive attitude towards PCC, both among health care workers and women^48,49^ where 98% believed in the health benefits of PCC on pregnancy outcomes.

There is a recommendation for the establishment of a special PCC clinic.^50^ A dedicated PCC clinic was established in the Netherlands, Hungary and the United Kingdom^51^ for the provision of PCC, but this is not a norm in Africa as some African countries like South Africa have opted for the integration of PCC services into already existing care.^43^ Interestingly, in the present study, most respondents (45.7%) disagreed that a dedicated clinic for PCC was a luxury service for women. Preconception care services can be rendered in a variety of settings. In the hospital settings, it has been recommended to be in the form of an opportunistic offer to every reproductive-aged woman encountered by health care providers. Nevertheless, the primary health care setting has been suggested to be the most suitable setting for these services.^50,52^ Similarly, almost half of the respondents (49.2%) had disagreed that the hospital setting was the best place to provide PCC. Globally, pregnancy planning hardly happens among the general population (World Health Organization);^20^ likewise, the unintended pregnancy rate in sub-Saharan Africa is higher than in the developed countries.^53^ Not surprisingly, in the present study, 47.1% had agreed that in their practice, population planning for pregnancy did not always happen.

Time has been cited in many studies as a major barrier to the provision of preconception services.^23,38,54^ It is noteworthy and surprising that about 58.7% of the respondents in the present study had disagreed that there was not enough time for the provision of PCC. Even though 71.7% of the present study respondents indicated that they had never received any form of training on PCC, yet 47.8% had disagreed that as a PHC nurse they did not have enough skills to offer PCC. Furthermore, a greater percentage (82.6%) had also disagreed that PHC nurses were not the best people to offer PCC. Studies have shown some degree of confusion about whose role it is to provide PCC and the need for PCC training for healthcare workers.^8,21,30,55^ In this study, 57.3% had disagreed that PCC without women asking for it, was unpleasant. This contrasted the study among health care providers in the United States,^23^ where it was revealed that providers shied away from talking about pregnancy desires with patients, except if patient initiated it themselves. Although in agreement with a study in the Netherlands,^30^ where a big percentage of respondents saw, raising of the topic about patients’ pregnancy wishes not a barrier to PCC.

## Strengths and limitations

The major limitation of the present study is that the data was collected using only the questionnaire, which may have limited the responses given by the respondents and may have been subjected to personal bias and the respondents’ capacity in interpreting the questions.

Another limitation is that the study was conducted in a higher education institution using only primary health care nurses who are students, which may not be generalised to the entire PHC nurses in the district.

## Implications

- There is a need to explore the patient’s knowledge and attitude of preconception care, especially in the context of HIV and AIDS.
- There is the need to explore the concept using the qualitative research method for in-depth understanding of the subject.
- Further studies can explore all the primary health care workers or all the health workers that are responsible for the provision of PCC.

## Conclusion

The results revealed that PHC nurses were knowledgeable about PCC, and they possess favourable attitudes towards PCC as well.

Knowledge and attitude were affected by the demographic characteristics. The knowledge of PCC was found to be influenced by study centre, employment area, and age. The high knowledge level among nurses in the rural area and those employed in the public clinics might be an indication of their job responsibility as majority of them initiate and manage patients on antiretroviral therapy. There is lack of PCC resources in the country. The provisions of PCC is very necessary for the improvement of pregnancy outcome; therefore, PHC nurses need to be prepared for this purpose.
